# Predictors and clinical complications associated with antiphospholipid antibodies in sickle cell disease

**DOI:** 10.1002/jha2.643

**Published:** 2023-01-13

**Authors:** Claudia Rodriguez Rivera, Andrew Srisuwananukorn, Rizma Jalees Bajwa, Victor R. Gordeuk, Joyce Rauch, Jerrold S. Levine, Santosh L. Saraf

**Affiliations:** ^1^ Department of Medicine Division of Nephrology University of Illinois at Chicago Chicago Illinois USA; ^2^ Department of Medicine Division of Hematology and Oncology Mt Sinai Health System New York City New York USA; ^3^ Department of Medicine Division of Hematology and Oncology University of Illinois at Chicago Chicago Illinois USA; ^4^ Department of Medicine Division of Rheumatology Research Institute of the McGill University Health Centre McGill University Montreal Quebec Canada; ^5^ Department of Medicine Division of Nephrology Jesse Brown, Veterans Affairs Medical Center Chicago Illinois USA

**Keywords:** antiphospholipid antibody, antiphospholipid syndrome, multiorgan failure, sickle cell disease, systemic lupus erythematosus

## Abstract

Although a higher prevalence of antiphospholipid autoantibodies (aPL) has been observed in some cohorts of sickle cell disease (SCD) patients, the clinical risk factors for the development of aPL and its associated complications remain unclear. In a retrospective study of 63 SCD patients, a lower hemoglobin concentration and higher white blood cell count were independently associated with an elevated aPL. SCD patients with elevated aPL had increased pregnancy complications (≥3 miscarriages, preterm delivery, pre‐eclampsia) and venous thrombotic events. Our findings suggest that SCD may predispose to the generation of aPL and that aPL itself may contribute to the vasculopathy of SCD. Prospective testing for aPL is warranted in patients with SCD.

## 1 INTRODUCTION

Antiphospholipid syndrome (APLS) is characterized by the presence of antiphospholipid autoantibodies (aPL) leading to thrombotic and pregnancy complications. Under several physiological and pathological situations, including hemolytic anemias, negatively charged phospholipids translocate from the inner to the outer surface of red blood cell (RBC) membranes, leading to the potential generation of an antigenic target for aPL. Indeed, an increased prevalence of aPL has been observed in patients with paroxysmal nocturnal hemoglobinuria or autoimmune hemolytic anemia, as compared to healthy controls [1, 2].

Sickle cell disease (SCD) is another hemolytic anemia affecting approximately 100,000 people in the United States and characterized by vasculopathy. Phosphatidylserine, a membrane phospholipid that is normally confined to the inner leaflet of RBC membranes, is expressed on the outer membrane of RBCs from SCD patients [3]. Although a higher prevalence of aPL has been observed in some cohorts of patients with SCD [4], the clinical risk factors for the development of aPL and their association with thrombotic or pregnancy complications remain unclear.

In a retrospective study of 63 adults with SCD who had aPL testing performed between August 1999 and June 2017, we tested the following two‐part hypothesis: 1) repeated exposure of the immune system of patients with SCD to the antigenic target of aPL leads to an increased formation of aPL; and 2) the presence of aPL is associated with pregnancy, thrombotic, and SCD‐related complications.

## 2 METHODS

The protocol was approved by the University of Illinois at Chicago (UIC) Institutional Review Board prior to undertaking the chart review. We included all SCD patients that had aPL testing performed as part of their routine medical care between August 1999 and June 2017. Laboratory and clinical data, including aPL levels, clinical diagnosis of systemic lupus erythematosus (SLE), SCD‐related complications, and pregnancy and thrombotic complications were extracted from the electronic medical record charting system, Cerner PowerChart (Cerner Corporation, Kansas City, MO, USA). Laboratory data were collected on the day closest to the time of aPL testing, during either the inpatient (*n* = 61) or the outpatient visit (*n* = 2). Vaso‐occlusive episodes were defined as pain episodes requiring medical attention (acute care center, emergency room visit, or inpatient hospitalization). Organ failure was defined as previously described [5]. Multiorgan failure was defined as the involvement of three organ systems simultaneously or within less than a week, and probable catastrophic antiphospholipid antibody syndrome (CAPS) was defined according to the international consensus statement as multiorgan failure in the presence of elevated aPL levels [6]. aPL testing (anti‐β2 glycoprotein I [anti‐β2GPI] IgM and IgG; anticardiolipin [aCL] IgM and IgG; and lupus anticoagulant [LA]) was performed in the UIC Clinical Pathology Laboratories as part of the patients’ routine medical care using Clinical Laboratory Improvement Amendments approved methods.

We compared individual aPL levels against categorical and linear variables using the Kruskal‐Wallis test and linear regression analysis, respectively. An elevated aPL was defined as any of the following: IgM or IgG anti‐β2GPI > 20 IU/ml, IgM or IgG aCL > 20 IU/ml, LA > 10 seconds. Clinical and laboratory variables were compared by elevated aPL status using the chi‐square test and Kruskal‐Wallis test, respectively. The association of independent variables with elevated aPL status was tested by multiple logistic regression analysis using a stepwise approach, adjusting for age, sex, and SLE status. Analyses were performed using Systat 13 (Systat Software Corporation; Chicago, IL, USA). Median values and interquartile ranges (IQRs) are provided.

## 3 RESULTS

**FIGURE 1:**
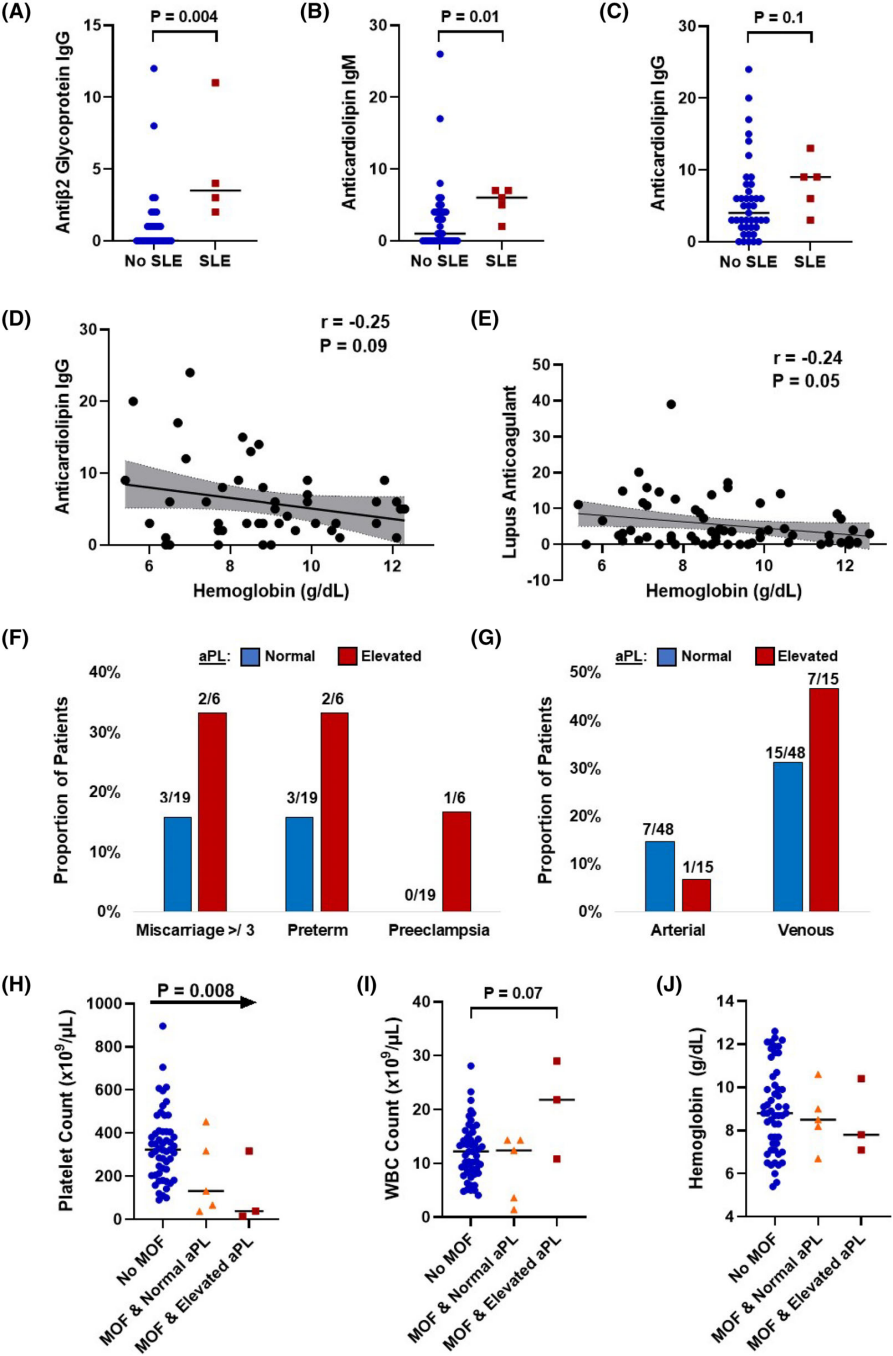
Association of antiphospholipid antibodies (aPL) with laboratory and clinical variables in patients with sickle cell disease (SCD). Patients with systemic lupus erythematosus (SLE) had higher (A) anti‐β2 glycoprotein I IgG and (B) anticardiolipin IgM levels and (C) a trend for higher anticardiolipin IgM levels compared to those without SLE. A lower hemoglobin concentration was associated with (D) a trend for higher anticardiolipin IgG levels and (E) the presence of a lupus anticoagulant. (F) Pregnancy complications and (G) venous thrombotic events were observed in a higher proportion of patients with an elevated aPL compared to those with a normal level. Blood counts in hospitalized SCD patients demonstrated (H) lower platelet and (I) a trend for higher white blood cell (WBC) counts in those with an elevated aPL and multiorgan failure (MOF), compared to those without MOF or MOF with a normal aPL; (J) no significant difference was observed for hemoglobin concentration

The median age of the SCD cohort was 32 years (IQR, 21–39 years), 49 (78%) were female, 62 (98%) were African American, five (8%) had a diagnosis of SLE, 43 (68%) were Hgb SS genotype, 17 (27%) were on hydroxyurea, and 16 (25%) were on chronic anticoagulation therapy. SCD patients on hydroxyurea had higher anti‐β2GPI IgG levels than patients not on hydroxyurea (2.5 vs. 0 IU/ml; *p* = 0.008); we did not observe significant differences in aPL levels by age, gender, or SCD genotype. SCD patients with SLE had higher anti‐β2GPI IgG and aCL IgM levels and a trend towards higher aCL IgG levels compared to those without SLE (Figure 1A–C). A lower hemoglobin concentration was associated with higher aCL IgG levels and prolonged LA (Figure 1D, E).

Fifteen of 63 (24%) patients with SCD had at least one elevated aPL. Patients with at least one elevated aPL versus no aPL had a lower hemoglobin concentration as well as trends towards a higher white blood cell (WBC) count, lower platelet count, higher total bilirubin concentration, and a lower estimated glomerular filtration rate (eGFR) (Table 1). A lower hemoglobin concentration (per 1 g/dl, odds ratio [OR] 1.74, 95% confidence interval [CI] 1.15–2.65; *p* = 0.009) and a higher WBC count (per natural log increment, OR 7.58, 95% CI: 1.29–45.45; *p* = 0.03) were independently associated with the presence of at least one elevated aPL, adjusting for age, sex, and prior SLE diagnosis.

**TABLE 1:** Patient characteristics by antiphospholipid (aPL) antibody status

	Normal (n = 48)	Elevated (n = 15)	P Value
**Age (years)**	29 (21 – 38)	29 (20 – 50)	0.8
**Females**	38 (79%)	11 (73%)	0.6
**Sickle Cell Genotype**			
**Hb SS or S** $\beta^{0}$ **‐thalassemia**	33 (69%)	11 (73%)	0.7
**Hb SC or S** $\beta^{+}$ **‐thalassemia**	15 (31%)	4 (27%)	
**Systemic lupus erythematosus (%)**	4 (8%)	1 (7%)	0.8
**Hydroxyurea (%)**	13 (27%)	4 (27%)	1.0
**Systolic blood pressure (mmHg)**	114 (108 – 130)	113 (96 – 123)	0.3
**Body mass index (kg/m^2^)**	23 (20 – 26)	24 (19 – 28)	0.9
**WBC count (x 10^3^/µL)**	11.8 (7.6 – 14.4)	13.5 (10.4 – 18.6)	0.06
**Hemoglobin (g/dl)**	9.0 (8.0 – 11.1)	7.7 (6.9 – 9.0)	0.01
**Platelet count (x 10^3^/µL)**	340 (224 – 406)	268 (122 – 317)	0.05
**Absolute reticulocyte count (x 10^3^/µL)**	192 (124 – 315)	195 (159 – 293)	0.7
**LDH (u/L)**	318 (243 – 474)	368 (265 – 506)	0.7
**Total bilirubin (mg/dl)**	2.2 (1.2 – 3.5)	2.8 (1.7 – 7.1)	0.09
**AST (u/L)**	39 (26 – 60)	44 (31 – 109)	0.3
**eGFR (ml/min/1.73m^2^)**	154 (115 – 208)	113 (17 – 157)	0.07
**Vaso‐occlusive episodes (preceding year)**	2 (0 – 3)	2 (1 – 4)	0.6
**Acute chest syndrome history (%)**	20 (42%)	9 (60%)	0.2
**Stroke history (%)**	9 (19%)	4 (27%)	0.5
**Priapism history (%)**	1 (10%)	1 (25%)	0.5
**Leg ulcer history (%)**	1 (2%)	0 (0%)	0.6

WBC, white blood cell count; LDH, lactate dehydrogenase; AST, aspartate transaminase; eGFR, estimated glomerular filtration rate.

Twenty‐five of 46 (54%) women had evaluable pregnancies; three additional women were excluded because of pregnancy outcomes that were not evaluable in our electronic medical charts. The presence of an elevated aPL was similar among women with a pregnancy (6/25 [24%]) compared to those who did not become pregnant (5/21 [24%]). Pregnancies complicated by ≥3 miscarriages, pre‐eclampsia, or preterm deliveries were more common in women with SCD who had an elevated (3/6 [50%]) versus non‐elevated (5/19 [26%]) aPL (Figure 1F).

Twenty‐nine of 63 (46%) SCD patients had a thrombotic event at the time of aPL testing. Of these, 23 were venous and eight were arterial (two patients had both a venous and arterial thrombotic event). The occurrence of a venous thrombotic event was more common among patients with (7/15 [47%]) vs. without (15/48 [31%]) an elevated aPL (Figure 1G). Arterial thrombotic events were less common in SCD patients with an elevated aPL level (1/15 [7%] vs. 7/48 [15%]).

Multiorgan multiorgan failure occurred in eight of the 61 (13%) hospitalizations. Three (20%) of the 15 patients with an elevated aPL met the criteria for probable CAPS. The SCD patients with probable CAPS had trends toward a lower platelet count (*p* = 0.05) and a higher WBC count (*p* = 0.07); similar hemoglobin concentrations were observed in SCD patients with and without probable CAPS (Figure 1H–J). The platelet counts progressively declined from SCD patients without multiorgan failure to those with multiorgan failure and a normal aPL and those with multiorgan failure and an elevated aPL (Figure 1H).

## 4 DISCUSSION

Previous SCD cohorts have demonstrated a high prevalence of elevated aPL, ranging from 8% to 66% [4, 7, 8]. Consistent with these reports, we observed an elevated aPL in 24% of SCD patients who had aPL testing performed. New in our study is the observation that anti‐β2GPI IgG and aCL IgM levels were higher in SCD patients with SLE. In addition, aCL IgG and LA times were inversely correlated with hemoglobin concentration. Notably, a lower hemoglobin concentration and higher WBC count were independently associated with an elevated aPL test. A lower hemoglobin concentration may signify worsening hemolysis and therefore greater antigenic exposure. A trend towards increased levels of hemolytic markers (lactate dehydrogenase, total bilirubin, and aspartate transaminase) was observed in SCD patients with an elevated aPL level, although the differences were not statistically significant. A higher WBC count reflects increased inflammation and predicts SCD severity [9]. Findings from our study are consistent with the hypothesis that the combination of hemolytic anemia and inflammatory stress synergize to promote the development of aPL in patients with SCD. This hypothesis warrants future investigation.

The development of aPL may impact the clinical course of SCD patients in two fundamental ways: by increasing their risk for typical APLS‐associated complications and/or by exacerbating the severity of their vaso‐occlusive episodes. Our findings suggest that both may occur. Pregnancy and thrombotic complications all trended higher in SCD patients with an elevated aPL. An increased risk for obstetric and thrombotic complications, hallmark features of APLS, has not been previously reported to the best of our knowledge. While the observed difference between venous and arterial events in our cohort is unexplained, it is known that the risk factors for venous versus arterial thrombotic events differ among patients with an elevated aPL [10]. In addition, the presence of an elevated aPL appeared to place SCD patients at greater risk for multiorgan failure. Triggering of a CAPS‐like state may occur not only via hemolysis and antigenic exposure but also via inflammatory stress, thereby providing the “second hit” thought to be necessary for systemic vascular complications [11]. If our findings are validated in further studies, aPL testing should be included in the routine workup of SCD patients, especially those with pregnancy or thrombotic complications.

There are several limitations to our findings, especially the retrospective nature of the study and its small sample size. The small sample size limits our ability to perform sensitivity analyses, such as by SLE status, SCD genotype, individual aPL test results, or types of thrombosis. Although the prevalence of an elevated aPL in 24% of our cohort was similar to that observed in the literature, there may have been a testing bias for aPL. Also, as most patients were tested while inpatient, there is the additional potential for selection bias. Both potential biases limit our ability to assess the association of aPL with thrombosis and pregnancy complications. Data on the etiology of increased hemolysis during hospitalization (e.g., auto‐immune hemolysis) and the presence of concurrent infections were not available but should be included in future studies. The aPL values that we analyzed represent a single assessment. We were unable to distinguish transient from persistent aPL; repeat measurements should be evaluated in future prospective studies. Future studies should also investigate the risk of developing aPL based on baseline clinical and laboratory variables. Despite these limitations, we observed a pattern of association between the presence of an elevated aPL and several adverse clinical outcomes suggesting a pathophysiologic role of aPL in the vasculopathy of SCD. Larger and prospective studies are needed to assess the prevalence and associated risk of elevated aPL in SCD patients, as well as to determine the mechanisms responsible for the generation of aPL in these patients.

## AUTHOR CONTRIBUTIONS

Claudia Rodriguez Rivera, Andrew Srisuwananukorn, Rizma Jalees Bajwa, Jerrold S. Levine, and Santosh L. Saraf designed and performed the research, analyzed the data, and wrote the paper. Victor R. Gordeuk and Joyce Rauch designed and performed the research and wrote the paper.

## CONFLICT OF INTEREST

The authors declare that they have no conflict of interest.
